# Implementing active surveillance for low-risk thyroid carcinoma into clinical practice: collaborative recommendations for Latin America

**DOI:** 10.20945/2359-4292-2023-0371

**Published:** 2024-06-03

**Authors:** Alvaro Sanabria, Carolina Ferraz, Chih Hao Chen Ku, Rosalia Padovani, Karen Palacios, José Luis Paz, Alejandro Roman, Anabella Smulever, Fernanda Vaisman, Fabian Pitoia

**Affiliations:** 1 Universidad de Antioquia Facultad de Medicina Departamento de Cirugía Medellín Colombia Departamento de Cirugía, Facultad de Medicina, Universidad de Antioquia, Medellín, Colombia; 2 Centro de Excelencia en Enfermedades de Cabeza y Cuello Medellín Colombia Centro de Excelencia en Enfermedades de Cabeza y Cuello (CEXCA), Medellín, Colombia; 3 Irmandade da Santa Casa de Misericórdia de São Paulo Divisão de Endocrinologia Departamento de Medicina São Paulo SP Brasil Divisão de Endocrinologia, Departamento de Medicina, Irmandade da Santa Casa de Misericórdia de São Paulo, São Paulo, SP, Brasil; 4 Universidad de Costa Rica Departamento de Farmacología Clínica y Toxicología San José Costa Rica Departamento de Farmacología Clínica y Toxicología, Universidad de Costa Rica, Clínica Los Yoses, San José, Costa Rica; 5 Clínica Diagnóstica Especializada VID División de Endocrinología Medellín Colombia División de Endocrinología, Clínica Diagnóstica Especializada VID, Medellín, Colombia; 6 Universidad Nacional Mayor de San Marcos Hospital Nacional Edgardo Rebagliati Martins Facultad de Medicina Lima Perú División de Endocrinología, Hospital Nacional Edgardo Rebagliati Martins, Departamento de Medicina, Facultad de Medicina, Universidad Nacional Mayor de San Marcos, Lima, Perú; 7 Universidad de Antioquia Hospital Universitario San Vicente Fundación Facultad de Medicina Medellín Colombia Sección de Endocrinología, Departamento de Medicina, Facultad de Medicina, Universidad de Antioquia, Hospital Universitario San Vicente Fundación, Medellín, Colombia; 8 Universidad de Buenos Aires Hospital de Clínicas División de Endocrinología Buenos Aires Argentina División de Endocrinología, Hospital de Clínicas, Universidad de Buenos Aires, Buenos Aires, Argentina; 9 Universidade Federal do Rio de Janeiro Instituto Nacional do Câncer Faculdade de Medicina Rio de Janeiro RJ Brasil Serviço de Oncoendocrinologia, Serviço de Endocrinologia, Faculdade de Medicina, Instituto Nacional do Câncer (Inca), Universidade Federal do Rio de Janeiro, Rio de Janeiro, RJ, Brasil

**Keywords:** Thyroid carcinoma, active surveillance, decision making, ultrasonography, risk, patient preference

## Abstract

The incidence of thyroid cancer is increasing globally, but mortality rates have remained steady. Many patients with thyroid cancer have low-risk, nonmetastatic intrathyroidal tumors smaller than 2 cm. Active surveillance has shown benefits in these patients, but the adoption of this approach remains below standard in Latin America. The purpose of this article is to identify ways to improve the incorporation of active surveillance into clinical practice for patients with low-risk thyroid carcinoma in Latin America, taking into consideration cultural and geographic factors. Current recommendations include three steps involving patient participation. The first step, which consists of the initial clinical examination, has eight factors requiring special attention. Anxiety must be managed while considering individual, disease-related, cognitive, and environmental aspects. Terms like "overdiagnosis", "incidentaloma," and "overtreatment" must be explained to the patient. Implementing precise terminology contributes to adequate disease perception, substantially reducing stress and anxiety. Clarifying the nonprogressive nature of thyroid cancer helps dispel myths surrounding the disease. The second step includes advice about procedures and guidelines for patients who choose active surveillance. Flexible monitoring techniques should be implemented, with regular check-ins scheduled based on patient needs. Reasons for adjusting treatment must be clearly communicated to the patient, and changes in preference regarding active surveillance should be considered in advance. The third step includes assistance during follow-up. Patients must be educated about ultrasound results and receive surgical indications from specialized physicians. The effectiveness of active surveillance can be reinforced by explaining to the patients the dynamics of changes in nodule size using clear and concise visual aids.

## Introduction

In recent decades, the incidence of thyroid cancer has increased globally, while the mortality rate has remained stable (1). This trend extends to the countries in Latin America (2,3). Currently, most thyroid tumors diagnosed in developed countries are intrathyroidal, nonmetastatic tumors smaller than 2 cm (4), classified as low-risk thyroid carcinomas.

Only a few studies have assessed the incidence of thyroid cancer in Latin America. In a study comparing the incidence of thyroid cancer in São Paulo (Brazil) versus that reported in the SEER database (United States), Veiga and cols. (5) described similar patterns in terms of age at diagnosis and tumor histology. Considering findings from surgical series indicating that 60%-75% of thyroid tumors measure less than 2 cm (6-9), it becomes evident that there is a considerable number of patients with low-risk thyroid carcinoma in Latin America who could potentially benefit from active surveillance. Still, the acceptance rate of active surveillance is lower in Latin America than in other world regions. Specifically, the acceptance rate is approximately 30% in Argentina, 10%-80% in Brazil, and below 20% in other Latin American countries. In contrast, it may reach 75% in developed nations like Canada and excees 90% in Japan (10-14). Notably, groups employing active surveillance for low-risk thyroid cancer in Latin America (11,12,15-18) have reported outcomes comparable to those reported in Japan (19), Korea (20), the United States (21), and Italy (22).

Qualitative studies suggest that certain factors influence the choice of treatment for low-risk thyroid cancer. These factors include the physician's perspectives (23), specialty and preferences (11), strategy adopted by the physician in presenting alternative treatments to the patient (24), type of information given to the patient (25), cultural and religious beliefs surrounding the disease (26), and idiosyncrasies (27). Latin American countries occupy a vast territory and comprise diverse populations (some with significant indigenous, African, or European ancestry) but share sociocultural characteristics that unite them (*e.g.*, Catholic religion, Latin-based language, Iberian influence). This unification results in attitudes and ideas that distinguish the countries in Latin America from those in other parts of the globe and must be considered when therapeutic options like active surveillance are proposed. According to Granda-Cameron (27), cultural values that influence the decision-making process in Latin America include "familialism" (*i.e.*, the patient's family is considered the primary source of support in the presence of illness and decision making on health care issues) and "simpatia" (*i.e.*, need for positive interpersonal interactions and desire to talk about the disease with others and to have a support network).

Given these facts, the experience of leading groups of thyroid specialists may help establish the minimum parameters required for improving the adherence of patients who are candidates for active surveillance, thus increasing physicians’ implementation of this treatment approach in Latin America. This document provides an outline of how active surveillance as an alternative treatment should be presented in Latin American nations, considering particularities specific to developing countries and limitations of health care systems in the region. The proposal is divided into three steps based on the moment of contact with the patient (Table 1).

**Table 1:** Recommendations for active surveillance in low-risk (Bethesda V-VI) thyroid nodules

Steps	Concepts	Recommendations
Step 1. Control of anxiety and psychological stress	Choose correct and precise words	Use "nodule with intermediate or high suspicion of carcinoma" or "low-risk carcinoma"
	Explain how the tumor was found and how it relates to the symptoms	Incidental finding or incidentaloma = asymptomatic Clinical finding = symptomatic Meaning of overdiagnosis, overtreatment, and "retrospective" active surveillance
	Explain if ultrasound and biopsy are not needed	Ultrasound is not required to investigate pharyngeal globus, hypothyroidism, or overweight Biopsy is not required for nodules smaller than 1 cm
	Ensure clarity	Describe the TI-RADS method and the likelihood of cancer (Table 2)
		Describe the Bethesda classification system and the likelihood of cancer (Table 3)
	Show the natural history of thyroid tumors considered to have a mild or high risk of malignancy	5%-11% of individuals who die of causes other than thyroid disease have undetected, asymptomatic thyroid cancer
	Explain alternatives options	Present alternative options fairly, considering how well they work, risks or side effects, and need for thyroid hormone therapy (Table 4) Begin with active surveillance
		Use whole numbers instead of percentages to convey the information, for example: "After 30 years of follow-up, 93 out of 100 patients will still have a stable nodule. There are three reasons why 7 out of the 100 patients will require surgery: (1) lymph nodes will grow or produce suspicious nodes in 3 cases; (2) the patient will change their mind in one case; and (3) the physician will change their opinion in 3 cases."
	Explain why the patient would benefit from active surveillance	Identify the patient's profile (i.e., ideal, appropriate, or inappropriate) (Table 5)
	Talk about things that could change treatment choices	Invite family or caretakers to take part in the decision-making process and talk about their concerns
		Recommend to the patient to avoid talking to others about treatment choices
		Make it clear that physicians have different points of view Establish follow-up at the same center and with the same group of professionals who made the initial recommendations
		Follow-up assessments must be done with discipline and be available to everyone
		Inform patients that they may change their minds at any time
Step 2: Explanation of how active surveillance works	Explain how often surveillance will occur	Clinical and ultrasound assessments should be performed every 6-12 months; it is currently unclear when these assessments should be interrupted
	Explain how tumors grow	Not all tumors behave the same way (Figure 1)
	Identify the reasons why surgery should be done	Nodule increases in size by more than 3 mm Smaller changes are considered variations by the examiner or the ultrasonography team Changes in how the tumor looks (*e.g.*, it expands outside the gland or becomes close to the trachea) Presence of suspicious lymph nodes Patient's decision
		Patients should be informed that they may change their minds at any time
	Identify the most likely type of surgery	Explain when lobectomy or total thyroidectomy is needed, and when neck dissection must be performed
Step 3: Maintenance of active surveillance	Explain in detail the terms used in ultrasound reports and what they mean for experts	How the growth of the nodule is estimated Reason for so many differences in measurements Most suspicious ultrasound findings Types of symptoms that the nodule could cause
	Show individual experiences of different patients	Display the tumor growth chart

**Table 2:** Risk of malignancy according to the TI-RADS classification (modified from Reference 76)

TI-RADS classification	Risk of malignancy
TI-RADS 1	<2%
TI-RADS 2	<2%
TI-RADS 3	2.1%-5%
TI-RADS 4	5.1%-20%
TI-RADS 5	>20%

**Table 3:** Risk of malignancy according to the Bethesda classification (modified from Reference 43)

Bethesda classification	Risk of malignancy
Bethesda 1	13% (5%-20%)
Bethesda 2	4% (2%-7%)
Bethesda 3	22% (13%-30%)
Bethesda 4	30% (22%-34%)
Bethesda 5	74% (67%-83%)
Bethesda 6	97% (97%-100%)

**Table 4:** Frequency of complications associated with each treatment alternative for low-risk thyroid carcinoma (modified from Reference 77)

	Active surveillance	Lobectomy	Total thyroidectomy
Need for surgery	5%-30%	100%	100%
Type of surgery	Lobectomy (50%), total thyroidectomy (50%)	Lobectomy (80%), total thyroidectomy (20%)*	Lobectomy (0%), total thyroidectomy (100%)
Long-term survival	>98%	>98%	>98%
Absence of cervical lymph node metastases	95%	95%	95%
Absence of distant metastases	>99%	>99%	>99%
Recurrent laryngeal nerve injury	0%	2%-5%	2%-5%
Tracheostomy	0%	0%	0.5%-2%
Definitive hypoparathyroidism	0%	0%	2%-7%
Hematoma	0%	<2%	<2%
Scar	No	Yes	Yes
Follow-up	>10 years	5-10 years	5-10 years
Frequency of visits	6-12 months	6-12 months	6-12 months
Need for thyroid hormone treatment	<10%	50%-60%	100%
Costs	$	$$$	$$$

* Intraoperative findings may change preoperative decisions.

**Table 5:** Recommendations for selecting patients for active surveillance (modified from Reference 62)

Candidate	Tumor	Patient	System
Ideal	Solitary Well-defined margins Surrounded by >2 mm parenchyma No extrathyroidal extension N0/M0	Aged >60 years Accepts active surveillance Understands the concept of salvage surgery Is disciplined Has a supportive family Has severe comorbidities	Multidisciplinary team Availability of ultrasound for follow-up Data collection Active monitoring
Appropriate	Multifocal Location far from recurrent laryngeal nerve/trachea Undefined margins Multiple nodules/thyroiditis Positron emission tomography (PET) positive	Aged 18-59 years Has family history of cancer Has a desire for pregnancy	Endocrinologist/surgeon in favor Ultrasound available
Inappropriate	Aggressive cytology Proximity to/invasion of recurrent laryngeal nerve/trachea Extrathyroidal extension N+/M+	Young age (<18 years) Disagrees with active surveillance	Inexperience Lack of conviction Concern about medicolegal issues Intolerance to uncertainty Reluctant scientific societies

**Figure 1:**
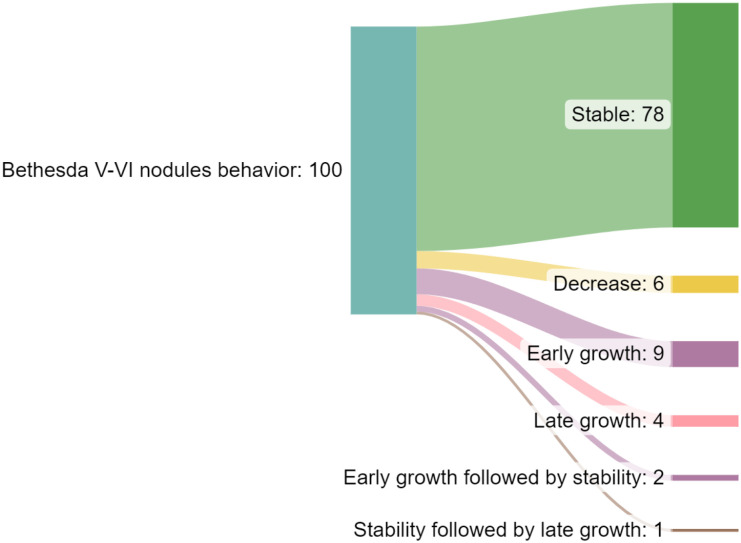
Changes in thyroid tumors during follow-up (modified from Reference 75)

## First step: initial clinical assessment

The purpose of the first step is to evaluate the imaging findings of the thyroid nodule in the context of the patient's clinical and sociocultural circumstances. At this point, most patients have contacted other health care professionals and received some information about the disease. They may also have obtained information from social media or websites and formed a vague understanding of the disease and its treatment (28). However, the information collected by the patients at this stage is not always reliable, and their opinion is frequently influenced by personal bias and information quality (29,30). In an intention-to-treat analysis, up to 50% of Latin American physicians opposed active surveillance, while the other 50% were receptive but did not offer it regularly to their patients (31). Furthermore, anxiety can limit the patient's ability to understand information and is often the most influential factor in their decision making (32). The following are eight major factors that must be addressed in this step.

### Managing anxiety and psychological tension

Key elements in models of anxiety in the face of cancer include individual factors (*e.g.*, personal or family history of cancer, intolerance of uncertainty), disease-inherent factors (perception of cancer as a life-threatening disease, uncertainty about the course of the disease), cognitive factors (beliefs and disease representation), and contextual factors (health care system and personnel, available information, social support) (33). Anxiety around the diagnosis of a thyroid nodule with potential for malignancy is linked to the information and emotional support received by the patient and to the types of treatment involved, although surgical complications and the need for thyroid hormone therapy are often overlooked in the process of treatment selection (34).

Significant factors triggering anxiety in patients include the amount and type of nonexpert information about treatment options, complications, prognosis, and disease follow-up collected before the appointment with the treating physician, the terminology used to refer to the clinical condition, pressure from the health care system and the society to diagnose and treat conditions preclinically (leading to overdiagnosis and overtreatment), influence from the medical culture (labeling, inadequate management of uncertainty, culture of blame when the diagnosis fail), and lack of knowledge by health care professionals (35). These factors should be presented explicitly at the beginning of the clinical evaluation to allow for a calm and moderate discussion and reinterpretation of the facts together with the patient.

Over 90% of the patients with thyroid cancer obtain information from various sources based on results from ultrasound (risk stratification according to ultrasound risk categories, *i.e.*, ATA or TI-RADS) and fine-needle aspiration biopsy (Bethesda system categories); however, only 53% of the sources that patients consult are reliable, and only half of the patients discuss the obtained information with their physicians (28,30,36). Studies show that over half of the information on websites, social media, and other open-access sources is inaccurate and misleading. Qualitative research has shown that many patients with Bethesda V and VI nodules who opt for surgery are unaware of the advantages of active surveillance (31). Since patients consider their treating physician a highly reliable source of information, the impact of misinformation should be discussed during the initial appointment (37).

### Clarifying the concepts of overdiagnosis, incidentaloma, and overtreatment

More than 50% of patients with an ultrasound finding of thyroid nodule had consulted a physician for conditions and symptoms unrelated to thyroid disease. Thyroid nodules found in patients with unsuspected thyroid disease are known as "incidentalomas." Studies show that most patients attribute the thyroid imaging results to their symptoms (38). Tests obtained for other disorders (*e.g.*, computed tomography imaging for follow-up of tumors at other sites) or unnecessary imaging (*e.g.*, ultrasound for hypothyroidism) result in more patients diagnosed with thyroid nodules (39), which is known as "overdiagnosis" (40). The probability of malignancy in a nodule incidentally detected on computed tomography imaging or ultrasound is minimal (11% and 2%, respectively). With overdiagnosis, additional investigation and therapies for the discovered nodule are implemented, resulting in "overtreatment" (41,42). These concepts must be explained to the patient so that symptoms truly caused by the nodule are identified and the consequences of unnecessary procedures are adequately explained.

### Explaining terms used for thyroid nodules

The terminology adopted in the Bethesda cytological classification system (43) may affect the patient's perception of the disease. Even though these terms have clear medical definitions, nonexperts sometimes overestimate the risk of cancer in noncancerous tumors (44). Physicians and patients sometimes mistake benign conditions for cancer due to the terminology used to describe both (*e.g.*, "neoplasia," meaning "new formation," and "atypia," meaning "infrequent finding"). Even the term "cancer" or "carcinoma" in the Bethesda V category may influence the patients’ views of their disease. The term used to explain alternative treatments has been shown to influence treatment decisions, since cancer calls for aggressive therapy. Using neutral designations can help patients make more informed decisions, reducing their worries and tension (34,45,46). To ensure patient comfort, it is important to use precise language during the interview. Some physicians use the Bethesda classification (nodule with a moderate or high risk of malignancy, along with its specific value) to explain the long-term clinical implications of the disease and the low likelihood of potential complications, while others use the term "carcinoma." The most important aspect is to explain the diagnosis to the patients and their families and friends and help them make informed treatment choices. Practical advice includes delaying treatment decisions for a few weeks and highlighting the fact that surgery is a "one-way" process, while active surveillance leaves room for future treatment possibilities, including surgery.

### Providing information on the natural history and prognosis of thyroid cancer

The Halsted model, which represents a gradual and predictable disease course, still dominates thyroid cancer research (47). However, it is important to note that prostate, breast, and thyroid cancers behave differently within this concept (48). Studies in Japanese cohorts undergoing active surveillance and followed up for more than 30 years have shown that most thyroid tumors remain stable over time, with a low risk of lymph node metastasis and rare cases of metastatic disease (8,19,49-51). This finding is also evidenced by the high prevalence of asymptomatic tumors discovered during autopsies. A recent retrospective Latin American study in patients with a single thyroid nodule reported similar routcomes among individuals who underwent active surveillance compared with those who initially had a benign cytological diagnosis that changed to papillary thyroid cancer after a second fine-needle aspiration biopsy or ultrasound surveillance obtained due to nodule growth (16). This information may help patients understand how thyroid tumors progress over time and appreciate that not all tumors require surgical removal.

### Coping of health care personnel with the concept of risk

Despite evidence against screening for thyroid nodules, some physicians still recommend screening when they are unaware of the natural history of low-risk thyroid cancer (39,52). Concerningly, fine-needle aspiration biopsy is sometimes obtained to investigate nodules smaller than 1 cm and with a low TI-RADS classification due to the physician's distorted perception of cancer risk and despite advice against this approach (53).

Health care providers also often make recommendations that are not founded on the probability of cancer. The TI-RADS approach, which uses ultrasound features to predict malignancy, is sensitive but has limited specificity (54), indicating that imaging findings are not always reliable to indicate cancer. The Bethesda classification system (43) is often mistakenly considered an indicator of disease stage, leading to biased interpretation and favoring aggressive treatment.

An unfavorable cultural perception of cancer is sometimes linked to the "war on cancer" concept (26,55). The metaphor of a fight centered on a pugnacious argument has proven problematic for health care professionals. This promotes "winners and losers," "victory and surrender," and disease as the "enemy." When the disease recurs or worsens, physicians and patients become fatalistic, terrified, and guilty, resorting to more aggressive treatments that may be unnecessary to the type of tumor (56).

Physicians’ personal beliefs have also been inadequately addressed when active surveillance is considered as therapy (57). Some physicians prefer surgery due to the heterogeneity of scientific recommendations, the complexity of considering multiple factors in treatment selection, structural difficulties related to the health care system, and even personal reasons like previous cancer experiences (58).

Finally, physicians and patients may be "minimalists" and prefer to do as little as possible while believing that many conditions are manageable without medical intervention or with natural treatments. Other physicians may be "maximalists" and seek treatment aggressively, and some others may be technology enthusiasts and early adopters of the latest medications or procedures for thyroid cancer treatment (59).

### Providing precise and reliable information about treatment alternatives

Physicians treating patients with low-risk thyroid nodules with potential for malignancy must provide them with concrete and balanced information about long-term efficacy, complications, and clinical outcomes of each of the three current treatment options (*i.e.*, active surveillance, surgery, and thermal ablation). The information provided to the patient should consider geographic-related factors (*e.g.*, availability of surgeons and devices). Most patients on active surveillance who are referred to surgery require lobectomy, but a study (17) has shown that surgeons often choose a more aggressive treatment (*e.g.*, total thyroidectomy), increasing the risk of complications. Of note, data have shown that salvage thyroidectomy is not associated with an increased risk of complications or recurrence in patients selected adequately for active surveillance (16).

The use of thermal ablation to treat low-risk thyroid nodules has been explored in recent studies. This type of therapy, still uncommon in Latin America, has been associated with results equivalent to those with surgery (60). In properly selected patients who are ideal candidates for lobectomy but reject this surgical approach, thermal ablation may be a viable alternative.

The order and structure in which information is presented to the patients may favor them to choose one therapy over another, as patients usually prioritize the first option presented to them. Following the heuristic theory of Kahneman & Tversky, active surveillance should be the first option presented to the patient (61). Additionally, a presentation with a positive outcome (survival rate) allows the patient to assess the situation more objectively than one with a negative outcome (recurrence rate).

The physician should justify the choice between lobectomy and total thyroidectomy. Many patients with low-risk thyroid carcinoma may be appropriately treated with lobectomy, but the patient should be informed that some intraoperative findings (*e.g.*, suspicious extrathyroidal invasion or undetected lymph node metastases) may change the type of surgery. A typical reason for total thyroidectomy is multinodular disease. Pathological features suggesting that tumors may migrate from low-moderate risk to high risk require radioiodine ablation, and up to 10% of patients may require a second surgery after lobectomy.

### Providing decision support

#### Clinical considerations for treatment decisions

Successful implementation of active surveillance requires the availability of some conditions related to the health care system, including the presence of a multidisciplinary team of surgeons and/or endocrinologists, high-resolution ultrasound, and organized follow-up structure. The treating physician must communicate to the patient the pros and cons of each treatment choice during the decision-making process. This discussion should be guided by the proposal delineated by Brito and cols. (62) of categorizing patients as ideal, appropriate, or inappropriate candidates.

The evaluation should include the assessment of the nodule's characteristics, including the risk of tracheal invasion and the angle between the suspicious nodule and the trachea (63). Compared with an acute angle, an obtuse angle suggests close contact between the tumor and the trachea, translating into increased risk of invasion. Of note, active surveillance should not be recommended for patients with tracheal cartilage invasion.

The most critical considerations from a clinical perspective are age and presence of severe comorbidities or diseases with a high surgical risk. Due to a higher growth rate of nodules at a young age, patients younger than 40 years are better candidates for surgery, whereas those older than 60 years are more suitable for active surveillance (64). Additionally, active surveillance is preferable for individuals with several comorbidities and increased surgical risk. Young women who wish to become pregnant in the short term or who are pregnant at diagnosis require special care. This population benefits from active surveillance despite not being "ideal" or "eligible" candidates for this treatment approach due to their age (65).

Regarding personal factors, surgical treatment is an option for individuals who are anxious and fearful about their diagnosis and want surgery promptly. Voice professionals and individuals who wish to avoid thyroid hormone treatment and surgery should choose active surveillance. Without imposing pressure, the physician must identify whether the patient has a "minimalist" or "maximalist" tendency. A practical approach is to suggest active surveillance without making it a final choice while considering the patient's feelings, how often they think about their condition, and how anxious and stressed they were in the week leading up to the appointment. These reactions can predict the patient's adherence to the active surveillance program.

Using graphical tools or other instruments to facilitate the patient's interpretation of the data increases their probability of understanding their treatment choices. Several authors have evaluated the use of such instruments (24,66,67). However, this approach is not always required, and its usefulness can be assessed for each patient (68).

#### Factors that can potentially modify treatment decisions

The decision-making process is dynamic and subjected to change over time. Physicians must consider several factors before initiating active surveillance on a patient with thyroid cancer, including the following:

- Consensus within the family regarding treatment decisions. Medical professionals and patients should discuss their decisions with the patients’ closest support group. The perception of the patients’ partners or caregivers is vital to active surveillance. Younger patients who rely more on their partners’ emotional support during stressful situations (*e.g.*, medical appointments) may not entertain active surveillance as a viable option. Due to family history and previous experiences, family members or caregivers may convince senior patients who choose active surveillance to switch to another therapy and vice-versa (69). Physicians may sometimes recommend that patients refrain from discussing sensitive information with their social circles. This could be due to religious or personal beliefs that such discussions could cause harm to the patient, or physicians may feel that the public's lack of understanding about thyroid cancer could impact the patient's well-being.
- Professional environment related to treatment options. Many experienced physicians advocate active surveillance for low-risk thyroid cancer, but few actually implement it (68). Patients requiring multiple specialists to manage other nonthyroidal conditions should be informed about the heterogeneity of thyroid nodule treatment and receive suggestions to modify their decisions. This applies to thyroid cancer specialists (*e.g.*, endocrinologists, surgeons) and physicians with whom patients have close ties (*e.g.*, family physicians) or incidental contact (*e.g.*, radiologists) (70-72).
- Educational and cultural status. Despite the preconceived notion that patients with higher socioeconomic status are less willing to accept active surveillance given their increased access to alternative treatments, some of these patients actually prefer this treatment approach. Due to their greater accessibility to resources and monitoring, patients with higher socioeconomic status may accept active surveillance when offered (73).
- Access to the health care infrastructure. In developing nations, access to health care services is crucial. Active surveillance works best with long-term monitoring and easy access to imaging testing. Lobectomy may be preferable for patients living in rural areas with limited access to medical care and those who face logistical challenges in accessing health care (*e.g.*, restrictions or variations in medical consultation and access to ultrasound specialists). It is important to note that patients who choose to undergo surgery may also face problems. For example, there are risks involved in being treated by a surgeon with limited experience in performing thyroid surgery. This may lead to an increased risk of complications or result in procedures that are more extensive than necessary (e.g., total thyroidectomy instead of lobectomy). To improve access to health care, a timeline has been tested to facilitate appointment scheduling and ultrasound imaging in various public services in Brazil. Cost-effectiveness should also be considered in the decision. According to local data, the costs of immediate surgery are three times greater than those of active surveillance over 10 years. Thus, implementation of active surveillance may help reduce health care disparities in the region (74).

## Considering decision changes

Patients must understand that they may change their treatment decisions without consequences to their relationship with the physician or to the prognosis of the disease. Up to 30% of the procedures in patients who choose active surveillance are performed due to patients’ decisions unrelated to the tumor (50). This circumstance should not be viewed as a failure of the initial treatment choice but rather as an integral part of the process. A change in decision from active surveillance to surgery is a scenario that should be discussed during the initial conversations and at each medical appointment. Additionally, physicians should be receptive to patients changing decisions and be careful not to make them feel guilty or regretful about that.

## Second step: selection of active surveillance

Once a decision for active surveillance is made, the patient should be guided through the therapeutic process. Most protocols offer detailed information on active surveillance, which must be tailored to the patients and their health care system. The information must be clearly explained to the patients to allow them to make necessary adjustments to adhere to the active surveillance scheme.

- Clinical and imaging monitoring should be performed every 6-12 months depending on the characteristics of the nodule (more frequently in higher TI-RADS categories), Bethesda classification (more frequently in higher categories), and assessment of the patient's degree of adherence (more frequently in patients with higher anxiety level). Monitoring should ideally be performed by the same physician or treating group, to avoid the adoption of different criteria, and using the same ultrasound equipment, given the expected variations in nodular measurements with different equipment.
- Reasons for change in treatment strategy from active surveillance to surgery should be determined clearly at the beginning. These include a >3 mm increase in the nodule's main diameter, change in the nodule's characteristics (appearance of irregular margins, extrathyroidal extension), or appearance of suspicious central or lateral cervical lymph nodes. Although the increase in nodular volume can be calculated, this should not be an absolute indication for surgery, which could lead to overtreatment.
- Change in opinion during active surveillance is a common indication for surgery and can occur soon after the decision for this therapeutic approach. This situation must be made clear, and the patient should have the option to undergo surgery if desired. The treating physician must embrace the patient's decision without causing sorrow or embarrassment.

## Third step: follow-up

The follow-up step consists typically of two periods: an "early period," in which the patients require a great deal of explanation and support from their physicians while facing an unfamiliar situation often viewed as counterproductive (monitoring a potentially malignant tumor), and a "later period," in which the patients’ concerns and doubts dissipate gradually until they find a calmer and more balanced emotional state and can understand precisely the goals of active surveillance (1,65). Even in the absence of evidence of growth or abnormal ultrasonographic changes, 10%-28% of patients who choose active surveillance change their minds and opt for surgery at some point during follow-up. A key cause for this shift is the sharing of information within the patients’ closest social circle (family and friends). Therefore, in addition to gaining the patients’ trust, the physician must also prioritize gaining their inner circle's trust (14,50).

During follow-up, it is crucial to emphasize several points:

1. Clarify the meaning of findings that may appear on follow-up images. The ultrasound report is a frequent source of anxiety for the patient during follow-up. It is also important to interpret specific technical terms in the report, such as extrathyroidal invasion or vascularization in the context of the nodule's behavior.
2. Recall the clinical indications for surgery and the potential for a shift in opinion regarding active surveillance.
3. Illustrate the behavior of the nodule size over time, preferably in a graphical form, so that the patient can trust the efficacy of the strategy.
4. Warn about the existence of different criteria for the management of thyroid nodules and the likelihood of obtaining inaccurate or biased information about the efficacy of the strategy.
5. Reiterate the lack of strong evidence regarding the use of thyroid hormone suppression of TSH levels.

In conclusion, the recommendations presented herein may serve as a guide for clinicians treating patients with thyroid nodules with a moderate or high degree of suspicion for whom active surveillance is an option. While most recommendations are based on scientific information, some derive from clinical practice experience and constant exposure to the patients’ concerns and questions. These recommendations are not rigid and can be adapted, modified, or discarded based on the preferences of the health care professional.
